# The Modern Technology of Radiation Oncology – Volumes I (1999) and II (2005)

**Published:** 2009

**Authors:** N. Suntharalingam

**Affiliations:** Emeritus Professor of Medical Physics, Department of Radiation Oncology, Thomas Jefferson University, Philadelphia, Pennsylvania, USA. Email: sunthasiva@verizon.net

These two volumes are a compendium of modern day technologies used in the practice of Radiation Oncology. Volume I, published in 1999, has 25 Chapters, written by a collection of 49 authors, who have all made significant contributions in the development of the discipline. Jake Van Dyk, as editor, has undertaken a daunting task and has proved his merit by accomplishing his goal of producing in one book, a valuable resource for the practicing professionals in the field. Volume II was published in 2005, as indicated by the editor, to cover the significant advances in new technologies, made since the publication of Volume I. These are well covered in 10 Chapters, again authored by 22 well-respected leaders in the field, who have successfully embraced these new technologies in their own departments. Jake van Dyk, as editor, has again admirably succeeded in having the authors remain focused in carefully describing the new technologies and how best to put them into clinical use. The two volumes cover a total of about 1500 pages. At the end of each chapter there is an extensive collection of references. The total number of cited publications in the two volumes is a very impressive collection of over 2800 articles.

This book is primarily written for Medical Physicists and Radiation Oncologists who are directly involved in the clinical implementation and utilization of new technologies for radiation treatment of different cancers. The chapters are all well written and do provide useful up-to-date information on the procedures, namely assessment of equipment needs, purchase, acceptance, commissioning, and quality assurance, that need to be followed for the successful implementation of new treatment approaches in the clinic. The book should also benefit several support staff, like dosimetrists (treatment planners), radiation therapists (technologists), computer scientists, and administrators, who all play significant roles in maintaining a standard of excellence in the delivery of radiation treatments.

Volume I spans over almost the entire field of Radiation Oncology, as practiced in the late 1990s. Most of the chapters present the material adequately. The chapters on Treatment Planning Systems and Dose Measuring Systems have a wealth of useful information and will be of much help to practicing clinical physicists, even if they do not belong to large academic centers. If there is one criticism, this reviewer would have liked to see an expanded coverage on IMRT in Chapter 12, which addresses Intensity Modulation, since IMRT was already in use in several centers. It was surprising to see a much larger coverage on Tomotherapy, even though it is highly unlikely that the use of such technology will be embraced in large numbers. Chapters 20 - 25, which describe some tested, but less used technology, although of scientific interest and curiosity, will have little impact on most clinically active treatment centers.

Volume II is a useful addition to Volume I and all the chapters are relevant to the modern day practice of computer-augmented treatment delivery. The chapter on Calibration of Megavoltage Radiation Beams should have been included in Volume I, as there have been very little advances to the methodology since the most recent protocols from the *American Association of Physicists in Medicine* (AAPM) and IAEA. Again, all chapters are well written and the technical details are presented in a clear and concise manner.

This book, both Volumes I and II, should definitely find a place in the shelves of all academic Radiation Oncology Centers and Libraries of Medical Schools worldwide. As intended by the editor, it is a useful addition as a reference text and also as an educational tool, primarily for medical physicists, graduate students, and clinical residents in medical physics. This reviewer is of the opinion that though the book is also written for use by Physicians (Radiation Oncologists and Residents in Radiation Oncology); it is highly unlikely that it will receive wide readership from such an audience. The physics and technology of the topics covered in Volume II are more demanding.

The total cost, in US Dollars, of the two Volumes is on the high side, especially for those working in the developing countries and even in countries like China and India. It would be helpful, and the book would be beneficially used by a much larger audience, if the editor together with the publisher, make suitable arrangements to publish this two volume book at a low cost, in a country like India.

